# Long-lasting and fast methylglyoxal-scavenging peptide CycK(Myr)R_4_E alleviates chronic pain in type 2 diabetic mice

**DOI:** 10.1097/PR9.0000000000001312

**Published:** 2025-08-12

**Authors:** Paramita Basu, Diogo F. S. Santos, Nina Gakii, Margaret R. Gralinski, Ryan B. Griggs, Sebastian Brings, Thomas Fleming, Keiichiro Susuki, Bradley K. Taylor

**Affiliations:** a Department of Anesthesiology and Perioperative Medicine, Department of Pharmacology, Pittsburgh Center for Pain Research, and Pittsburgh Project to End Opioid Misuse, University of Pittsburgh School of Medicine, Pittsburgh, PA, USA; b Department of Neuroscience, Cell Biology, and Physiology, Boonshoft School of Medicine, Wright State University, Dayton, OH, USA; c Department of Medicine I and Clinical Chemistry and the Department of Nuclear Medicine, University Hospital Heidelberg, Heidelberg, Germany; d Department of Medicine I and Clinical Chemistry, University Hospital of Heidelberg, INF 410, Heidelberg, and the German Center for Diabetes Research (DZD), Neuherberg, Germany; e Department of Pharmacology and Chemical Biology, University of Pittsburgh, Pittsburgh, PA, USA; f Center for Neuroscience at the University of Pittsburgh, Pittsburgh, PA, USA

**Keywords:** CycK(Myr)R_4_E, db/db, Diabetes, Methylglyoxal, Methylglyoxal scavenger, Painful diabetic neuropathy

## Abstract

**Introduction:**

Pathological levels of methylglyoxal (MG), a reactive dicarbonyl product of glucose, contribute to major neurological complications associated with type II diabetes, including chronic neuropathic pain. Strategies to target elevated MG have included small molecule MG scavengers, but scavenger deficiencies in proteolytic stability and onset of scavenging activity have precluded clinical translation. To address this gap, we developed a long-lasting and highly reactive cyclic peptide CycK(Myr)R_4_E, and here evaluated its antihyperalgesic efficacy in the db/db mouse model of type II diabetes painful diabetic neuropathy.

**Objectives:**

To test the hypothesis that CycK(Myr)R_4_E can reduce behavioral and molecular signs of painful diabetic neuropathy.

**Methods:**

We assessed heat hypersensitivity as an index of hyperalgesia, and touch-evoked expression of phosphorylated extracellular signal–regulated kinase as a measure of neuronal activity in spinal cord dorsal horn.

**Results:**

We report that a single systemic injection of CycK(Myr)R_4_E (3 mg/kg) reversed heat hypersensitivity. Repeated systemic injection of CycK(Myr)R_4_E (0.125 mg/kg, 3 times per week, 6–12 weeks of age) prevented heat hypersensitivity and reduced stimulus-evoked phosphorylated extracellular signal–regulated kinase.

**Conclusion:**

These studies promote CycK(Myr)R_4_E as the most promising MG scavenger for the prevention and treatment of hyperalgesia in type 2 diabetic neuropathic pain.

## Abstract 2

Supplemental Digital Content is Available in the Text.

CycK(Myr)R_4_E, a novel methylglyoxal scavenger with superior proteolytic stability and prolonged circulation time, reduced behavioral and molecular signs of painful diabetic neuropathy in mice.

## 1. Introduction

High blood glucose levels and insulin deficiency are hallmarks of type 2 diabetes mellitus (T2D).^12^ Adverse pathophysiological consequences of T2D include metabolic disorders, vascular damage, organ damage, and neurological complications associated with peripheral neuropathy.^2,16,27^ A particularly debilitating feature is the neuropathic pain of painful diabetic neuropathy (PDN),^37^ with an alarming lifetime prevalence that is particularly high in women.^25^ The debilitating symptoms of PDN include spontaneous pain, hyperalgesia, and allodynia, all of which are resistant to currently available analgesic drugs,^38,53^ and so new treatments are desperately needed.

The underlying mechanisms that govern PDN include disturbances related to inflammatory factors, hyperglycemia, lipid metabolism, and oxidative stress.^24,52–54^ Of particular interest to the latter is methylglyoxal (MG), the most abundant reactive dicarbonyl byproduct of glucose in patient plasma. MG covalently modifies proteins, leading to production of advanced glycation end-products (AGEs). Blood levels of MG and AGEs correlate not only with the development of diabetic nephropathy, diabetic neuropathy, and diabetic retinopathy^3,4,7,8,20,30^ but also pain. Indeed, diabetic patients with PDN present with elevated levels of plasma MG compared to patients without PDN.^11^

Methylglyoxal depolarizes sensory neurons in vitro, and cutaneous administration of MG causes pain-like behaviors including cutaneous hypersensitivity to mechanical, heat, and cold stimuli in mice^11,14,59^–this occurs in humans as well through direct activation of C-nociceptors.^23^ Furthermore, deletion mutant mice lacking glyoxalase (GLO1, an enzyme that metabolizes MG) exhibit heat and mechanical hypersensitivity that is associated with elevations in MG in plasma, DRG and sciatic nerve and elevations in MG-AGEs in serum.^11^ These data support the translational potential of treating PDN with strategies to normalize elevated circulating levels of MG. One such strategy is scavengers that form peptide-MG adducts. Many small molecule scavengers have been developed, but clinical translation has been hindered by a short half-life (creatine, free arginine, and pyridoxamine^13,43,56^), slow-scavenging activity (metformin^9,10,36^) that does not prevent the formation of human serum albumin -MG-H1 adduct in vitro (metformin and pyridoxamine^14^), and/or side effects in clinical trials (aminoguanidine^14,29^).

CycK(Myr)R_4_E is a novel arginine-rich, fatty acid–coupled, cyclic peptide. Each of the arginine side chains of CycK(Myr)R_4_E reacts with a molecule of MG and removes it from the blood. Coincubation of metformin and CycK(Myr)R_4_E resulted in a similar activity as the incubations of CycK(Myr)R_4_E alone, which confirms the additive effect of CycK(Myr)R_4_E to metformin.^14^ In addition, in vitro biodistribution and pharmacokinetics of CycK(Myr)R_4_E are more favorable as compared to GERP_10_, especially from a translational perspective.^14^ Unlike GERP_10,_ cyclization contributes to increased plasma stability, while the attachment of myristic acid improves peptide half-life most likely through noncovalent binding of albumin. Also, CycK(Myr)R_4_E generates MG-modified peptide adducts that undergo efficient renal excretion.^14^

We reported that CycK(Myr)R_4_E rapidly decreased plasma levels of MG and MG-AGEs for long periods of time in vivo and also decreased acute nociception produced by intravenous administration of MG.^14^ To determine whether the pain-relieving actions of CycK(Myr)R_4_E extend to the chronic hyperalgesia associated with PDN, we tested single and repeated administration protocols in db/db mice.

## 2. Methods

### 2.1. Animals

All experiments were conducted in accordance with guidelines from the International Association for the Study of Pain^61^ and with the approval of the Institutional Animal Care and Use Committees of the University of Pittsburgh, the University of Kentucky, and the Wright State University. C57BL/6J mice (Jackson Laboratories, Bar Harbor, ME) or db/db mice and their controls (Jackson Laboratories, Bar Harbor, ME) were housed at a maximum of 4 males or 5 females per cage in a temperature- and humidity-controlled room with food and water available ad libitum on a 14-hour light 10-hour dark cycle with lights on from 6:00 am to 8:00 pm. We restricted behavioral studies to 8:00 am–6:00 pm to avoid steep changes in physiological rhythms surrounding lights-on or lights-off transitions. db/db mice and their genetic db/+ controls were fed standard chow. We acknowledge that hormonal changes during the estrous cycle can influence pain in women. However, we used females without consideration of their stage in the estrous cycle based on the consensus report of the Sex, Gender, and Pain Special Interest Group of the International Association for the Study of Pain, which states that “The value of testing female rodents at different stages of the estrous cycle is debatable,” in part because “an influence of estrous cycle stage is not necessarily indicated by larger observed variance” and because “measurement of the estrous cycle would require daily handling, and the ensuing stress could affect nociception or sensitivity to drugs.”^31^

### 2.2. Mouse model of type II diabetes

Diabetic db/db mice (BKS.Cg-Dock7m+/+Lepr^db^/J) are homozygous for the diabetes spontaneous mutation (Lepr^db^), and this manifests as morbid obesity, chronic hyperglycemia, pancreatic beta cell atrophy, and hypoinsulinemia. We used normoglycemic db/+ mice (BKS.Cg-Dock7m +/+ Lepr^db^/J, commonly referred to as C57BLKS/J, or BKS^17^ as genetic controls for db/db mice. To initiate our colony at the University of Pittsburgh, we interbred db/+ with C57BL/6 background purchased from Jackson Laboratories (Bar Harbor, ME) to expand our colony of db/db + genotypes. These were paired to generate db/db mice and their littermate controls.

### 2.3. Drug administration and timelines

After measuring the predrug baselines of all mice within each strain of an experiment, we randomly assigned them into groups of similar baseline values.

#### 2.3.1. Systemic methylglyoxal

For systemic MG administration, cages of male and female C57BL/6J mice (The Jackson Laboratory; RRID:IMSR_JAX:000664) aged 6 to 8 weeks were divided into vehicle control and MG groups. MG (M0252, Sigma) dissolved in saline at a concentration of 50 mg/kg body weight was administered intraperitoneally 5 days a week from day 1 to day 25.

**Figure 1.**
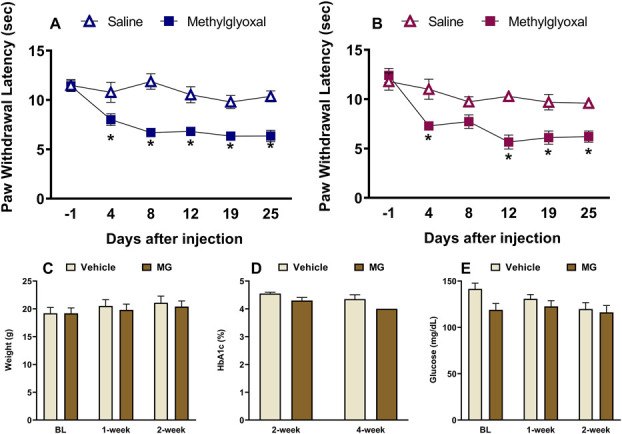
Repeated systemic administration of methylglyoxal (MG) (50 mg/kg body, i.p.—5 days a week from D1-D25) increases heat sensitivity in male (A) and female (B) mice. n = 6 per sex. Repeated MG administration did not change weight (n = 8 per group) (C), HbA1c level (n = 3–4 per group) (D), or blood glucose levels (n = 8 per group) (E) in either sex. Data represented as mean ± SEM. (*P* > 0.05). *P < 0.05 saline vs methylglyoxal (MG).

#### 2.3.2. Systemic CycK(Myr)R_4_E

CycK(Myr)R_4_E or saline was administered to unanesthetized male and female db/db and db/+ mice by the intraperitoneal (i.p.) route either as a single injection (0.3 or 3.0 mg/kg) at 7 to 8 weeks of age or by repeated injection (0.125 mg/kg) 3 times per week for 6 weeks (6–12 weeks of age).

**Figure 2.**
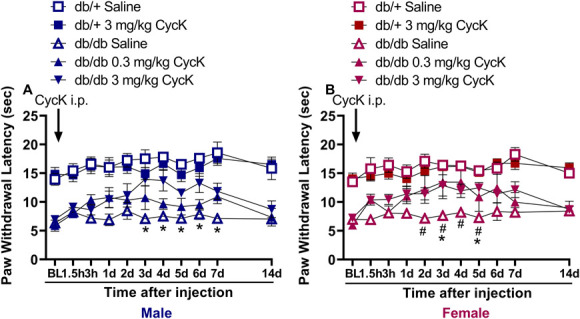
Single intraperitoneal injection of CycK(Myr)R_4_E (CycK) (0.3 and 3 mg/kg) dose-dependently reversed heat hypersensitivity in the (A) db/db mouse model of T2D, but not in db/+ (heterozygous controls). Effects began within hours and lasted several days. (B) Area under the curve (AUC) analyses revealed CycK(Myr)R_4_E reduced heat hypersensitivity in db/db mice compared to the db/+ (heterozygous controls). n = 9 to 13 per group. Data represented as mean ± SEM. (*P* < 0.05). (A)–Two-way ANOVA; (B)–One-way ANOVA. *P < 0.05 db/db CycK(Myr)R_4_E 3 mg/kg vs db/db saline. #*P* < 0.05 db/db CycK(Myr)R_4_E 0.3 mg/kg vs db/db saline.

**Figure 3.**
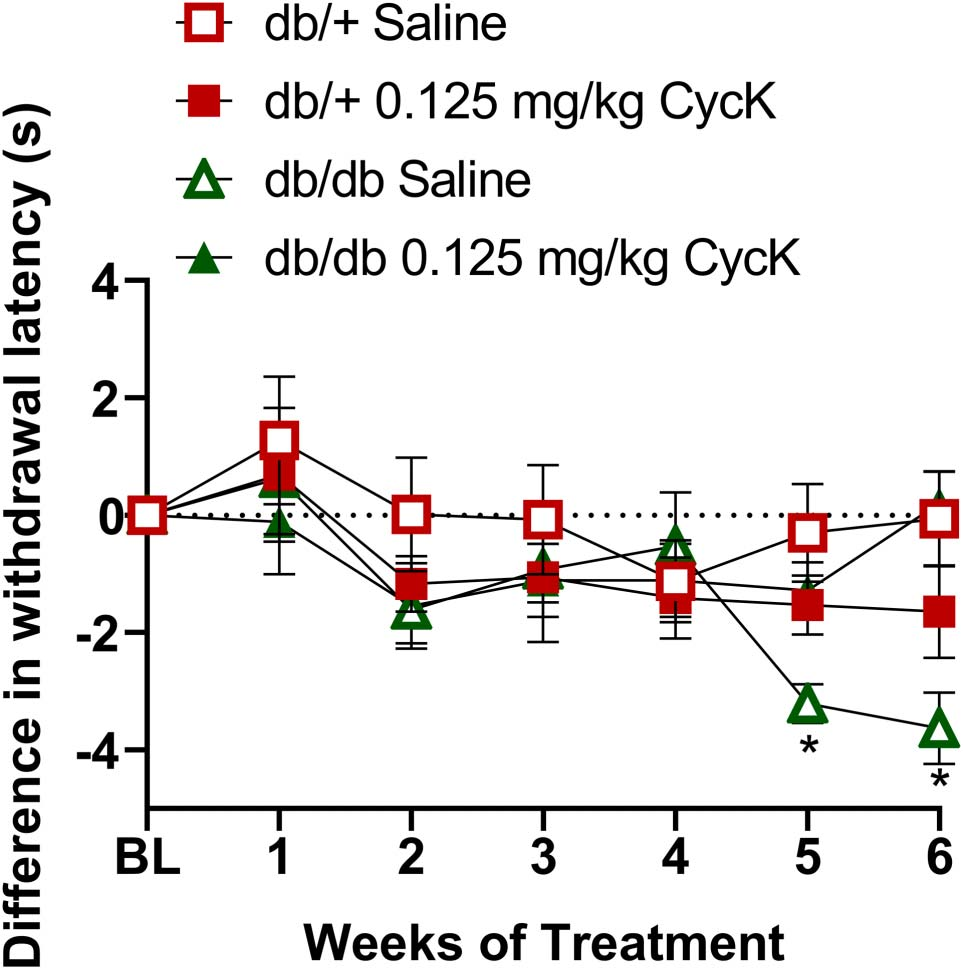
Repeated intraperitoneal injections of CycK(Myr)R_4_E (CycK) attenuated heat hypersensitivity in db/db mice. Line graph describing heat sensitivity at the hind paw after the systemic administration of CycK(Myr)R_4_E (0.125 mg/kg, i.p.) in db/db and db/+ (heterozygous controls) mice. n = 7 to 8 per group, males and females aggregated. Data represented as mean ± SEM. *P < 0.05 db/db CycK(Myr)R_4_E vs db/db saline.

#### 2.3.3. Intrathecal CycK(Myr)R_4_E

In awake mice, CycK(Myr)R_4_E or saline was injected in both sexes at 7 to 8 weeks of age with escalating doses by the intrathecal route of administration (5 µL) as follows: one 10 µg dose, followed 2 days later with 2 doses of 30 µg, followed 2 days later with 2 doses of 100 µg (Suppl Fig. 1, http://links.lww.com/PR9/A330).

### 2.4. Measurement of blood glucose and hemoglobin A1c

A small incision was made on the tip of the tail to obtain a drop of blood. Blood glucose and hemoglobin A1c (HbA1c) were measured by collecting a drop of blood onto either a TrueMetrix blood glucose test strip (Trividia Health, Fort Lauderdale, FL) or the A1CNow + blood collection cartridge (PTS Diagnostics, Indianapolis, IN) (Fig. 1).

### 2.5. Hotplate assay of heat sensitivity

As described previously,^34^ heat sensitivity was assessed by placing db/db or db/+ mice on a heated surface (52.5 ± 1°C) within an acrylic enclosure (Hotplate; Columbus Instruments, Columbus, OH) followed by measurement of the time to hind paw escape (eg, jumping, licking, or flinching of either hind paw). Paw withdrawal latency at each timepoint was calculated as the average time to response across 3 trials per animal, with an intertrial interval of approximately 3 minutes. Animals were immediately removed after paw response or a cutoff of 25 seconds to avoid tissue injury. The animals were acclimated to the testing environment and hotplate apparatus before experimental manipulations by placing them one at a time in the hotplate enclosure for 5 minutes with the hotplate turned OFF (room temperature) on day 1, and the hotplate turned ON on days 2 and 3 before drug administration.

#### 2.5.1. Timelines

For the repeated systemic MG administration study in C57BL/6J mice, hotplate testing was conducted on days −1 (one day before starting MG administration), baseline (BL), 4, 8, 12, 19, and 25 (Fig. 1). For the single systemic drug administration study in db/db mice, hotplate testing was conducted at 1.5 hours, 3 hours, 1 day, 3 days, 4 days, 5 days, 6 days, 7 days, and 14 days (Fig. 2). For the repeated systemic CycK(Myr)R_4_E studies in db/db mice, hotplate testing was conducted once weekly for a total of 6 weeks (Fig. 3). For intrathecal CycK(Myr)R_4_E studies, hotplate testing was conducted at BL, 0.5 hours, 1.5 hours, and 3 hours after injection (Suppl Fig. 1, http://links.lww.com/PR9/A330).

### 2.6. Phosphorylated extracellular signal–regulated kinase

After the final behavioral testing session (week 6), mice were lightly anesthetized with isoflurane (5% induction and then 1.5% maintenance), and the ventral surface of the left hind paw was mechanically stimulated for 2 seconds with a cotton swab with a gentle heel to toe stroke. This was repeated every 5 seconds for 5 minutes. After an additional 10-minute pause, mice were deeply anesthetized with isoflurane and transcardially perfused with cold 0.01-m PBS (Fischer Scientific) with added heparin sodium salt (VWR, NJ) (10,000 USP units/L), followed by 10% buffered formalin phosphate (Fisher Chemical). Lumbar spinal cords were harvested by dissection and postfixed in the same fixative overnight at 4°C and then cryoprotected with 30% sucrose until the tissue sank to the bottom of the polypropylene round-bottom tube (Becton Dickinson Labware, NJ) at 4°C (1–3 d).

**Figure 4.**
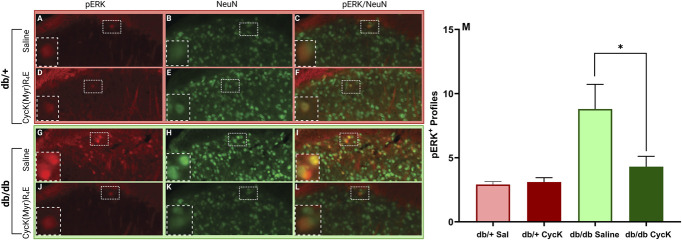
Repeated administration of CycK(Myr)R_4_E (CycK) reduced touch-stimulated pERK expression in the spinal cord dorsal horn on the side ipsilateral to simulation. (A–L) Representative images from mice treated with either saline (A–C in db/+ and G–I in db/db mice) or CycK(Myr)R_4_E (0.125 mg/kg, i.p.) (D–F in db/+ and J–L in db/db mice). Sections were colabeled with pERK (A, D, G, J) plus NeuN (B, E, H, K). Images were taken with a ×20 objective. Insets are magnified images of the boxed region to better show immunoreactivity of NeuN and pERK. (M) Histograms illustrating the number of pERK + profiles in lamina I–II. Images were taken with a ×10 objective. Scale bars: 100 μm. 3 to 4 mice per group. Data represented as mean ± SEM. *P < 0.05 db/db CycK(Myr)R_4_E vs db/db saline.

### 2.7. Immunohistochemistry

Transverse sections (35 µm) from L3–L5 segments were obtained with a sliding microtome (SM2000R, Leica). The sections were washed in 0.01-m PBS, blocked in 3% normal goat serum (GeminiBio) containing 0.3% Triton X-100 (Sigma-Aldrich) in 0.01-m PBS for 1 hour, and then incubated with rabbit primary antibody to phosphorylated-ERK1/2 (1:1000; catalog #4370S, Cell Signaling) and Alexa Fluor 488 mouse NeuN (1:500; catalog #MAB377X, EMD Millipore) at 4°C for 24 hours on a shaker (The Belly Dancer, Stovall Life Science, NC) at approximately 150 rpm. The following day, sections were washed in 0.01-m PBS and then incubated with goat antirabbit Alexa Fluor 568 (1:1000, catalog #A-11011, Invitrogen) for 1 hour at room temperature. Finally, the sections were washed in 0.01-m phosphate buffer, mounted onto slides (catalog #12255015, Fisherbrand), and then coverslipped with ProLong Glass Antifade mounting medium with DAPI (catalog #P36984, Thermo Fisher Scientific) (Fig. 4).

### 2.8. Imaging

All images (pERK- and NeuN-positive profiles) were captured in laminae I-II of the spinal cord dorsal horn with a Nikon TE-2000 using a 20× or 40× objective (numerical aperture 0.45) and analyzed using NIS-Elements Software AR analysis 4.13.05 Software (Nikon).

### 2.9. Blinding procedure

We employed blinding procedure for each experiment. One experimenter placed transgenic mice (db/db vs db/+) into test chambers and coded each tube for drug or vehicle, whereas another experimenter conducted behavioral testing. Codes were released at experimental completion. For image analysis, the investigator was blinded to experimental group throughout image acquisition.

### 2.10. Statistical analyses

Data were graphed and analyzed using Prism software (version 9.0, GraphPad, La Jolla, CA). The sample size was determined based on our previous publications using identical outcome measures.^6,45,46,48^ We designed our studies with the factors that included Time, Genotype, Drug, and Sex. It is important to note that these sample sizes were not large enough to power 3-way Time × Genotype × Drug ANOVA, but this experimental design achieves our goal to inform the power needed for future 3-factor studies. Two-factor ANOVA that included Drug × Time and Drug × Genotype and Drug × Sex were conducted. Drug × Sex and Drug × Genotype interactions were tested with Time collapsed across timepoints using the trapezoidal method for calculation of area under the curve (AUC). In Drug x Time repeated measures experiments, when the main effect was significant, differences at each timepoint were tested with Bonferroni post-hoc analyses. The average of 4 to 5 sections per animal were quantified for phosphorylated extracellular signal–regulated kinase (pERK) in laminae I-II. Statistical significance was set at *P* < 0.05. All data are presented as mean ± SEM.

## 3. Results

### 3.1. Repeated administration of methylglyoxal induced heat hypersensitivity in both sexes

Previous studies reported that single or repeated systemic administration of MG produced mechanical and heat hypersensitivity in male mice and rats; however, females were not considered.^11,14,59^ Figure 1 fills this gap and illustrates that repeated injection of MG to C57BL/6J mice produced heat hypersensitivity that persisted for at least 4 weeks in both males (*P* < 0.05, Table 1, line 1) and females (*P* < 0.05, Table 1, line 4). This occurred without a change in body mass (*P* > 0.05), HbA1c (*P* > 0.05), as well as blood glucose (*P* > 0.05) levels, suggesting no major effect on animal health.

**Table 1:** Statistical analyses.

Figs	Sex	Assay	Line #	ANOVA type	Factors	F	*P*
1a	M	Heat—HP	1	2-Way	Drug × time	(5, 60) = 3.68	<0.05
			2		Time	(5, 60) = 7.3	<0.05
			3		Drug	(1, 59) = 75.5	<0.05
1c	F	Heat—HP	4		Drug × time	(5, 60) = 3.92	<0.05
			5	2-Away	Time	(5, 60) = 11.8	<0.05
			6		Drug	(1, 60) = 52.14	<0.05
2a	M	Heat—HP	7	2-Way	Drug × time	(40, 253) = 0.84	>0.05
			8		Time	(10, 253) = 4.01	<0.05
			9		Drug	(4, 253) = 121.9	<0.05
2b	F	Heat—HP	10	2-Way	Drug × time	(40, 231) = 0.8	>0.05
			11		Time	(10, 231) = 4.2	<0.05
			12		Drug	(4, 231) = 99.31	<0.05
3a	M + F	Heat—HP	13	2-Way	Drug × time	(18, 182) = 1.14	>0.05
			14		Time	(6, 182) = 4.3	<0.05
			15		Drug	(3, 182) = 29.14	<0.05
3a	M + F	Heat—HP	16	3-Way	Time	(6, 182) = 4.29	<0.05
			17		Genotype	(1, 182) = 48.33	<0.05
			18		Drug	(1, 182) = 35.39	<0.05
			19		Time × genotype	(6, 182) = 0.68	>0.05
			20		Time × drug	(6, 182) = 0.77	>0.05
			21		Drug × genotype	(1, 182) = 8.87	<0.05
			22		Time × genotype × drug	(6, 182) = 1.99	>0.05
4m	M + F	pERK	23	1-Way	Drug	(3, 23) = 6.32	<0.05
S1a	M + F	Heat—HP	24	2-Way	Drug × genotype	(1, 13) = 0.01	>0.05
			25		Genotype	(1, 13) = 187.0	<0.05
			26		Drug	(1, 13) = 0.02	>0.05
S1b	M + F	Heat—HP	27	2-Way	Drug × genotype	(2, 17) = 0.65	>0.05
			28		Genotype	(1, 17) = 90.06	<0.05
			29		Drug	(2, 17) = 0.48	>0.05
S1c	M + F	Heat—HP	30	2-Way	Drug × genotype	(2, 14) = 2.31	>0.05
			31		Genotype	(1, 14) = 135.3	<0.05
			32		Drug	(2, 14) = 2.68	>0.05

1-Way, one way; 2-way, 2 way; 3-way, 3 way; F, female; HP, hotplate; M male; M + F, male–female combined; pERK, phosphorylated extracellular signal–related kinase.

### 3.2. Single systemic injection of CycK(Myr)R_4_E reversed heat hypersensitivity in db/db mice

Brings et al. reported that a single injection of CycK(Myr)R_4_E (0.25 mg/mouse, i.p.) in C57BL/6 mice decreased the acute nociception produced by intravenous MG (5 μg/g).^14^ Single injection of other small molecule MG scavengers such as aminoguanidine, alagebrium, d-arginine, or metformin^11,35^ reduced heat hypersensitivity and hind paw flinching in the streptozotocin model of type 1 diabetes. However, type 2 diabetes accounts for 90% of patients and is more frequently associated with PDN,^1^ and the mechanisms of diabetic neuropathy may differ in these 2 forms of diabetes.^15,28^ Therefore, to determine whether the actions of CycK(Myr)R_4_E extend to the chronic hyperalgesia associated with PDN, we studied db/db mice. As illustrated in Figure 2 and consistent with previous findings,^33,34^ db/db mice exhibited heat hypersensitivity. This was attenuated with CycK(Myr)R_4_E (0.3 and 3 mg/kg, i.p). A two-way Drug x Time ANOVA revealed CycK(Myr)R_4_E attenuated heat hypersensitivity in both males (*P* < 0.05, Table 1, line 9, main effect of Drug) and females (*P* < 0.05, Table 1, line 12, main effect of Drug) for up to 6 days postinjection as compared to db/+ controls.

### 3.3. Repeated systemic injection of CycK(Myr)R_4_E decreased behavioral and molecular signs of painful diabetic neuropathy

Repeated administration of an MG scavenger offers advantages over single administration by providing sustained protection against MG-induced toxicity, accumulation of AGEs, and cellular damage.^51^ In the current study, we tested the effects of repeated CycK(Myr)R_4_E administration on behavioral and molecular signs of PDN.

#### 3.3.1. Behavior

As illustrated in Figure 3, repeated administration of CycK(Myr)R_4_E attenuated heat hypersensitivity in db/db mice. A two-way Drug x Time ANOVA revealed that CycK(Myr)R_4_E attenuated heat hypersensitivity in db/db mice at week 6 (*P* < 0.05, Table 1, line 15; main effect of Drug) compared to db/+ controls. A three-way ANOVA (Drug × Genotype × Time) revealed a main effect of Genotype × Drug interaction (*P* < 0.05, Table 1, line 21).

#### 3.3.2. Stimulus-evoked phosphorylated extracellular signal–regulated kinase expression

Phosphorylated extracellular signal–regulated kinase in the spinal cord dorsal horn is an established marker for the activation of spinal nociceptive neurons and possibly central sensitization^39–41^ after peripheral nerve injury,^44^ inflammation,^18^ surgery,^6,19^ and in models of type 2 diabetes including ZDF rats,^32^ streptozotocin,^22,35,55^ intraplantar administration of MG,^33^ and db/db mice.^11,21,55,58^ We extended these studies to a repeated CycK(Myr)R_4_E administration protocol in db/db mice. As illustrated in Figures 4A–F, saline and CycK(Myr)R_4_E-treated db/+ mice exhibited minimal pERK expression in dorsal horn. Consistent with previous reports, we observed elevated pERK expression in db/db mice. Importantly, as illustrated in Figures 4G–L and 4M, repeated administration of CycK(Myr)R_4_E decreased pERK expression as compared to saline (*P* < 0.05, Table 1, line 23).

### 3.4. Intrathecal injection of CycK(Myr)R_4_E did not change heat hypersensitivity

The spinal cord dorsal horn has been suggested to contribute to the pronociceptive effects of MG.^34,42,57^ For example, Griggs et al. reported that intrathecal administration of the MG scavenging peptide GERP_10_ alleviated heat hypersensitivity in db/db mice for at least 120 minutes.^34^ To determine whether these effects extended to CycK(Myr)R_4_E, we administered it intrathecally and tested heat hypersensitivity. Supplemental Figure S1a, S1b, and S1c (http://links.lww.com/PR9/A330) illustrate the data with Time collapsed across the 30- to 180-minute timepoints (*P* > 0.05, Table 1, lines 24, 26, 27, 29, 30, 32), revealing no significant effect of CycK(Myr)R_4_E (10 µg, 30 µg, and 100 µg, i.t.) on db/db mice compared to saline (*P* > 0.05).

Our intrathecal CycK(Myr)R_4_E data remain inconsistent with a previous report indicating that intrathecal administration of the MG scavenging peptide GERP_10_ alleviated heat hypersensitivity in db/db mice.^34^ The differences could be attributed to the different targets of GERP_10_ and CycK(Myr)R_4_E at the spinal level, which warrants further investigation.

## 4. Discussion

### 4.1. Methylglyoxal maintains lasting heat hypersensitivity in both sexes

We report for the first time that repeated intraperitoneal administration of MG (50 mg/kg/d) induced a long-lasting heat hypersensitivity in both male and female mice. This extends previous reports of MG-induced mechanical hypersensitivity that were restricted to male mice. First, injections of MG (2.0 or 5.0 mg/kg/d, i.p.) on days 2, 4, 6, 8, and 10 produced nociception in male Sprague-Dawley rats^42^ or male ICR mice.^5^ Second, a single injection of MG (5 or 50 but not 0.5 μg, intravenous) elicited hypersensitivity to heat, mechanical, and cold stimuli that lasted up to 2 days in male C57BL/6 mice.^11,14,59^ Overall, these findings indicate that MG reliably causes hypersensitivity in both sexes and provide support for the use of chronic administration protocols to mimic and thus better understand the contribution of MG to chronic neuropathic pain in T2D.

### 4.2. A single injection of CycK(Myr)R_4_E provides very long-lasting relief from heat hypersensitivity

Consistent with the antinociceptive actions of diroximel fumarate^49^ and FEM-1689 after intraplantar injection of MG,^60^ we previously reported that a single injection of CycK(Myr)R_4_E decreased the acute nociception produced by intravenous MG.^14^ Here, we extend these findings to the db/db mouse model of T2D neuropathic pain and report that a single injection of CycK(Myr)R_4_E attenuated heat hypersensitivity that lasted for 6 days. This duration is longer than the 3 days of pain relief provided by GERP_10_ in our previous study,^34^ and this likely reflects the superior pharmacokinetic properties of CycK(Myr)R_4_E.^14^

### 4.3. Repeated CycK(Myr)R_4_E reduces heat hypersensitivity and spinal neuron activation

In the current study, we found that repeated treatment with CycK(Myr)R_4_E attenuated heat hypersensitivity in db/db mice. This is consistent with and extends previous reports indicating that repeated administration of other MG scavenging peptide, GERP_10_ (given on days 1, 3, and 6), aminoguanidine (days 1 and 2), or alagebrium (ALT-711) (days 1 and 2) ameliorated MG-evoked or streptozotocin-induced heat hyperalgesia in mice.^11^ Furthermore, we found that repeated treatment with CycK(Myr)R_4_E attenuated the expression of pERK, a marker of neuronal activation in the superficial laminae of the dorsal horn that mediates nociceptive transmission. These studies are reminiscent of previous studies showing that a ketogenic diet^26^ or administration of the ketone bodies acetoacetate or β-hydroxybutyrate^50^ increased glyoxalase 1 activity, decreased MG concentrations, and reduced MG-induced hypersensitivity and spinal pERK expression.

#### 4.3.1. Intrathecal CycK(Myr)R_4_E does not alleviate heat hypersensitivity

The current study found that intrathecal CycK(Myr)R_4_E did not alleviate heat hypersensitivity in db/db mice. Possible explanations for the inability of CycK(Myr)R_4_E to alleviate hypersensitivity at the spinal level could be as follows: (1) CycK(Myr)R_4_E is attached to a fatty acid (myristic acid), which theoretically makes the molecule more lipophilic than GERP_10_. This increased lipophilicity should increase passive diffusion across the dural membrane and into the spinal cord tissue faster and thus decrease the potency of CycK(Myr)R_4_E at the spinal level; (2) myristic acid was designed to enhance albumin binding and systemic stability of CycK(Myr)R_4_E. The turnover time for cerebrospinal fluid in mice is 1.8 hours.^47^ The strong albumin binding of CycK(Myr)R_4_E could reduce the free fraction available to diffuse into tissues.

## 5. Conclusion

We conclude that CycK(Myr)R_4_E reduces behavioral hypersensitivity and dorsal horn neuronal activation in the db/db mouse model of type II PDN. Either single or repeated injection of CycK(Myr)R_4_E alleviated heat hypersensitivity with extended duration in both sexes. Repeated injection was also associated with a decrease in stimulus-evoked pERK expression in the superficial dorsal horn, indicating that it works by decreasing the activity of spinal neurons that process and/or relay the transmission of noxious inputs to the brain. Our findings support MG scavengers as a promising pharmacotherapeutic strategy for the treatment of neuropathic pain associated with diabetes, and point to CycK(Myr)R_4_E as the most promising candidate of its class. This is due to its relatively high specificity and favorable pharmacokinetics that include superior stability, extended half-life, efficient renal excretion, and fast ability to scavenge methylglyoxal.

## Disclosures

The authors have no conflict of interest to declare.

## Supplemental digital content

Supplemental digital content associated with this article can be found online at http://links.lww.com/PR9/A330.

## Supplementary Material

SUPPLEMENTARY MATERIAL
