# Supplementary material for: Long-lasting and fast methylglyoxal-scavenging peptide CycK(Myr)R4E alleviates chronic pain in type 2 diabetic mice
Source: Pain Rep. 2025 Aug 12;10(5):e1312. doi: 10.1097/PR9.0000000000001312 (PMC12348387; doi:10.1097/PR9.0000000000001312)
Supplement: SUPPLEMENTARY MATERIAL [file painreports-10-e1312-s001.pdf]

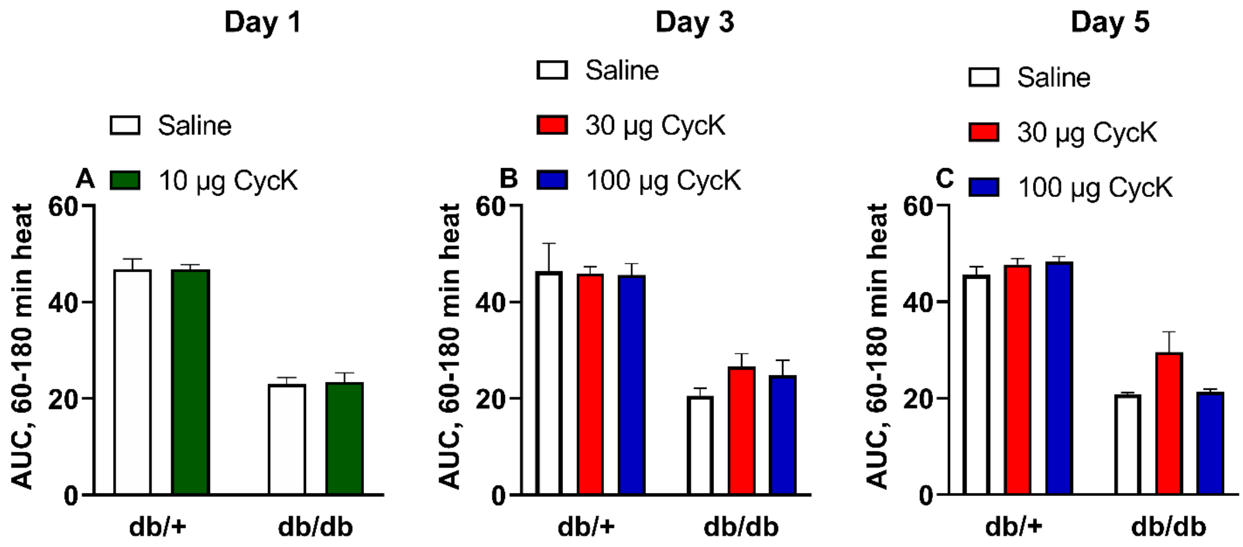

Suppl Figure 1. Intrathecal administration of CycK(Myr)R_4E did not attenuate heat hypersensitivity in db/db mice after (**A**) day 1 (n = vehicle - db/+ & db/db – 4; 10 µg Cyck - db/+ - 4, db/db – 5), (**B**) day 3 (n = vehicle – db/+ - 2, db/db – 4; 30 µg Cyck - db/+ - 4, db/db – 6; 100 µg Cyck – 4, db/db - 3), and (**C**) day 5 (n = db/+ & db/db - vehicle – 2; 30 & 100 µg Cyck - db/+ & db/db - 4) of treatment. Data represented as mean ± SEM. ($p>0.05$. Two-way ANOVA).
